# Hydrogelation of Regenerated Silk Fibroin via Gamma Irradiation

**DOI:** 10.3390/polym15183734

**Published:** 2023-09-12

**Authors:** Peerapat Thongnuek, Sorada Kanokpanont, Pimpon Uttayarat, Siriporn Damrongsakkul

**Affiliations:** 1 Center of Excellence in Biomaterial Engineering for Medical and Health, Chulalongkorn University, Bangkok 10330, Thailand; peerapat.t@chula.ac.th (P.T.); siriporn.d@chula.ac.th (S.D.); 2 Biomedical Engineering Program, Faculty of Engineering, Chulalongkorn University, Bangkok 10330, Thailand; 3 Biomedical Engineering Research Center, Faculty of Engineering, Chulalongkorn University, Bangkok 10330, Thailand; 4 Department of Chemical Engineering, Faculty of Engineering, Chulalongkorn University, Bangkok 10330, Thailand; 5 Research and Development Unit, Thailand Institute of Nuclear Technology (Public Organization), Nakhon Nayok 26120, Thailand; pimponu@tint.or.th

**Keywords:** silk fibroin, hydrogel, gamma radiation, gelation, chain scission, radiation crosslinking

## Abstract

Gamma irradiation, which is one of the more conventional sterilization methods, was used to induce the hydrogelation of silk fibroin in this study. The physical and chemical characteristics of the irradiation-induced silk fibroin hydrogels were investigated. Silk fibroin solution with a concentration greater than 1 wt% formed hydrogel when irradiated by gamma rays at a dose of 25 or 50 kGy. The hydrogel induced by 50 kGy of radiation was more thermally stable at 80 °C than those induced by 25 kGy of radiation. When compared to the spontaneously formed hydrogels, the irradiated hydrogels contained a greater fraction of random coils and a lower fraction of β-sheets. This finding implies that gelation via gamma irradiation occurs via other processes, in addition to crystalline β–sheet formation, which is a well-established mechanism. Our observation suggests that crosslinking and chain scission via gamma irradiation could occur in parallel with the β–sheet formation. The irradiation-induced hydrogels were obtained when the solution concentration was adequate to support the radiation crosslinking of the silk fibroin chains. This work has, therefore, demonstrated that gamma irradiation can be employed as an alternative method to produce chemical-free, random coil-rich, and sterilized silk fibroin hydrogels for biomedical applications.

## 1. Introduction

Silkworm silk derived from *Bombyx mori* has been used for centuries in biomedical applications, notably as suture materials, due to its biocompatibility, biodegradability, and decent mechanical properties. Silk fibers are spun from silkworm silk as a mix of two proteins, namely silk sericin and silk fibroin (SF). Silk sericin, which makes up about 20–30% of the silk weight, serves as an adhesive between SF polypeptides, allowing them to bundle up, thereby improving the strength of cocoons [1,2]. However, biomedical silk materials are preferably fabricated from SF without sericin due to its better biocompatibility. Evidence has shown that SF supports cell adhesion and growth. Furthermore, SF-based biomaterials elicit negligible immunogenic reactions [1,3,4]. The SF was approved by the FDA for use as a biomedical material in 1993 [1]. SF materials also possess adjustable biodegradability [3,4]. Their degradation rates can be tuned based on the types used and the numbers of crosslinks present. Hence, SF can be a candidate for use in biodegradation-dependent drug delivery systems. Several biomedical materials fabricated from SF have been made commercially available, such as tissue-regenerative scaffolds, Seriscaffold^®^, and SeriACL^TM^.

SF naturally functions as a structural molecule that gives shape and mechanical robustness. This protein is made of heavy (390 kDa) and light (25 kDa) chains that are bridged together by a disulfide bond [5]. While the light chains are relatively more hydrophilic and contain no apparent repetitive amino acid motif, the more hydrophobic heavy chains have repeats in their primary structures, including Gly-Ala-Gly-Ala-Gly-Ser, Gly-Ala-Gly-Ala-Gly-Tyr, and Gly-Ala-Gly-Ala-Gly-Val-Gly-Tyr. These repeats allow the formation of crystalline antiparallel β-sheets in the secondary structure. Many studies examined the relationships between the fraction of β-sheets and the strength and stability of SF-based biomaterials. They found that both the strength and stability mainly depend on the percentage of β-sheets [6]. Apart from the mechanically important β-sheets, other secondary structures, including β-turns, random coils, and α-helices, are less well studied in the field of SF heavy chains.

SF in a solution can spontaneously form hydrogel. It was previously established that spontaneous gelation starts with inter- and intramolecular weak interactions, such as hydrogen bonding, hydrophobic, and electrostatic interactions [5]. These interactions hold together SF polypeptides in solution and prompt them to make further changes when sufficient energy input is applied. However, at this stage, SF can scarcely change its secondary structures due to its metastability [5,7]. After a period in the solution, the SF chains rearrange themselves to form the thermodynamically stable crystalline β-sheets. The correlation between the increase in the fraction of β-sheets and the progress of gelation suggests that β-sheet formation is pivotal to the gelation process.

Various techniques were employed to fabricate SF hydrogels, the majority of which were thought to exert energy to accelerate the β-sheet formation [5,8]. SF gels faster when the solution temperature rises from room temperature to 37 °C [5]. This outcome was explained based on phase separation between the dissolved SF polypeptides and the surrounding ordered water molecules. The separation frees SF from interactions with water, allowing them to rearrange to form β-sheets [5]. Lowering pH dramatically decreases the gelation time. SF in acidic solution quickly gels at a pH lower than the pK_a_ of SF (4.5 for acidic side chains and 10 for alkaline side chains), and the relatively predominant carboxyl side chains are neutralized via protonation, while the less predominant amino side chains become charged. It was proposed that hydrophobic interactions overcome the repulsion between the charged amino side chains, thus accelerating the formation of the hydrophobic crystalline β-sheet structures [5]. At a roughly neutral pH, both carboxyl and amino side chains are charged. This fact causes electrostatic interactions between the opposite charges that result in inter- and intramolecular interactions, supporting β-sheet formation. At pHs above 10, the protonation of the predominant carboxyl side chains causes charge repulsion that hinders interaction between SF polypeptides. Interestingly, the fraction of β-sheets in the gel is unchanged, regardless of the pH at which the gel forms [5].

External energy, such as sonication, vortexing, electric fields, and gamma radiation, can also be applied to induce gelation. Sonication accelerates gelation by raising the local temperature to the extent that the SF hydrophobic domains are freed from the surrounding ordered water molecules and interact to form crystalline β-sheets [9]. Vortexing exerts shear stress on the SF molecules. The resultant shears are believed to increase the number of β-sheets in SF polypeptides by destroying the prevailing weak bonds, hence increasing the chance of reforming more thermodynamically stable β-sheets [10]. Also, SF-concentration fluctuation in a vortex results in an increase in the local concentration of the β-sheet motifs, which leads to the formation of crystalline β-sheet structures [10]. Gelation via the application of an electric field yields hydrogels with a slightly different proportion of secondary structures [7,11]. This type of hydrogel has a reduced fraction of random coils and an increased fraction of α-helices compared to spontaneously formed hydrogels. However, the fractions of β-sheets are not significantly different. The increase in the α-helices was shown to correlate with gelation, highlighting the role played by α-helices in the stability of the hydrogels.

Gamma irradiation, which has been conventionally used as a sterilization method, can also be applied to induce the crosslinking of polymer chains to form a three-dimensional network, thus enabling the gelation process [12,13,14,15]. This crosslinking process is mediated by free radicals generated during irradiation. This irradiation-induced crosslinking technique has been demonstrated in various polymeric hydrogel systems, including polyethylene, poly(vinyl alcohol), and other biopolymers [16,17,18,19]. The radiation helped the SF solution to more quickly gel, but the resulting hydrogels much more easily degraded [12]. The latter outcome implies that the hydrophobic crystalline β-sheet structures, which resist degradation, might be destroyed. Yet, the way in which the SF secondary structures are changed after gamma irradiation-induced gelation is still unknown. This study aimed to extend the knowledge of the structural changes in the SF solution after irradiation and explain the way in which the changes affect the stability of hydrogels. In the long run, gamma irradiation may become an alternative method of simultaneously fabricating and sterilizing fibroin hydrogels.

## 2. Materials and Methods

### 2.1. Materials

Silk cocoons of *Bombyx mori* Thai silkworms “Nangnoi-Srisaket 1” were produced at the Queen Sirikit Sericulture Center, Nakhon Ratchasima province, Thailand. All chemicals used were of laboratory or reagent grade. Gamma Chamber—GC 5000 with ^60^Co source (Thailand Institute of Nuclear Technology (Public Organization), Ongkharak, Nakhon Nayok) was used to irradiate the silk solutions at a dose rate of 4 kGy/h.

### 2.2. Preparation of Silk Fibroin Hydrogel

SF solution was prepared as previously reported [20,21]. In brief, silk cocoons were degummed via boiling for 20 min in 0.02 M Na_2_CO_3_ solution (Sigma-Aldrich, Singapore) and rinsed 5 times in deionized water. The degumming and rinsing steps were repeated once again with the same number of cycles. The degummed fibers were subsequently dried before being dissolved in 9.3 M LiBr solution (Sigma-Aldrich, Singapore) at 60 °C (16 mL LiBr: 4 g degummed fibres). The solution was then dialyzed for 3 days against deionized water (MWCO 12,000–16,000, Viskase Company Inc., Tokyo, Japan). The dialysate was centrifuged to remove debris. The resulting solution was adjusted to 1, 2, 4, or 7 wt% before being poured (7.5 mL) into test tubes (capped, 10 mL in size). The samples were subjected to gamma irradiation at the Thailand Institute of Nuclear Technology (Public Organization), Ongkharak, Nakhon Nayok province, Thailand. They were exposed to irradiation at doses of 25 or 50 kGy at the rate of 4 kGy/hour. Spontaneously formed hydrogels were prepared by leaving the SF solution in a closed chamber at 25 °C until gelled. The gelation was monitored via test-tube inversion. The solution was considered gelled when the bulk did not flow after inverting the test tube. The resulting SF hydrogels were characterized for gel fraction, thermal analysis using differential scanning calorimetry, and structure and conformation using Fourier transform infrared spectroscopy and X-ray diffraction techniques.

### 2.3. Insoluble Gel Fraction Test

The fibroin hydrogels were dried for 2 days in an incubator at 50 °C before being submerged for 24 h in deionized water at 37 °C. The hydrogels were then retrieved from the water, dried for 2 days in an incubator at 50 °C, and weighed. The insoluble gel fraction was calculated as follows:

(1) $$gel\;fraction\;\left(\%\right)=\frac{W_{Gf}}{W_{Gi}}\times 100$$

$W_{Gf}$ and $W_{Gi}$ represent the dry weights of the gels before and after submersion, respectively.

### 2.4. Free Amino Group Evaluation of Silk Fibroin

The amount of free amino groups present in SF hydrogels was determined using a 2,4,6-trinitrobenzene sulfonic acid (TNBS, Thermo Scientific, Waltham, MA, USA) assay, which was modified from those of Burnis, W.A. and Ofner, C.M. [22]. In brief, the lyophilized samples of SF hydrogels and solutions were treated with 1 mL of 4% *w*/*v* sodium hydrogen carbonate (NaHCO_3_, Ajax Finechem, Auckland, New Zealand) and 1 mL of 0.5% *w*/*v* TNBS solution at 40 °C. The samples were then hydrolyzed using 12 N of HCl (QReC, Queenstown, New Zealand) for at least 12 h until the clear solutions were obtained. The spectrophotometric measurements of the solutions at 415 nm were performed, and the number of free amino groups was calculated using the standard curve of the β-alanine solution (Nacalai, Kyoto, Japan). The experiment was performed in quadruplicate.

### 2.5. Thermal Stability Test

The hydrogels were cut, weighed, and incubated for 3 h at 80 °C in water. The remaining hydrogels were dried. After that stage, they were again weighed. The percentages of the remaining weights were calculated.

### 2.6. Transition Temperature Determination

The hydrogels were lyophilized and crushed. The transition temperatures of the crushed samples were then analyzed via the differential scanning calorimetry (DSC) technique (Mettler Toledo: model DSC 1 STAR System, Leicester, UK). The samples were subjected to a heating rate of 10 °C /min, with the temperature rising from 20 °C to 300 °C.

### 2.7. Structural and Conformational Analysis

The hydrogels were lyophilized and crushed prior to the structural and conformational analyses via the Fourier transform infrared spectroscopy (FTIR) and X-ray diffraction (XRD) techniques. For FTIR measurement (Thermo Scientific: model Nicolet 6700, USA), the crushed hydrogels were mixed with potassium bromide, and disc-shaped samples were prepared. FTIR Fourier self-deconvolution and the curve fitting of spectra in the amide I region (1575–1725 cm^−1^) were analyzed using Omnic 8.0 software. The amount of beta-sheet conformation was quantified from the areas under the assigned peaks of the 1616–1637 and 1696–1703 cm^−1^ regions. The tyrosine residue (1595–1615 cm^−1^), random coil (1638–1655 cm^−1^), alpha-helix (1656–1662 cm^−1^), and beta turn (1663–1696 cm^−1^) structures were also calculated from the areas of the defined peaks [23]. XRD analysis (Bruker: model D8 Advance, Leipzig, Germany) of crushed hydrogels was performed at a diffraction angle (2θ) of 5–40 degrees.

### 2.8. Statistical Analysis

The statistical analysis was performed on IBM SPSS statistics (version 22) and GraphPad Prism (version 7) using one-way ANOVA and Bonferroni’s post hoc tests or Tukey’s multiple comparisons test. In addition, two-way ANOVA, followed by Tukey’s multiple comparisons test, was also used. Differences were considered to be statistically significant when *p* ≥ 0.05.

## 3. Results and Discussion

### 3.1. Gelation of Silk Fibroin Solution

The effects of SF concentration and irradiation dose on the formation of hydrogels are presented in Table 1. Non-irradiated SF solutions slowly gelled within 4–6 days at 25 °C.

**Figure 1:**
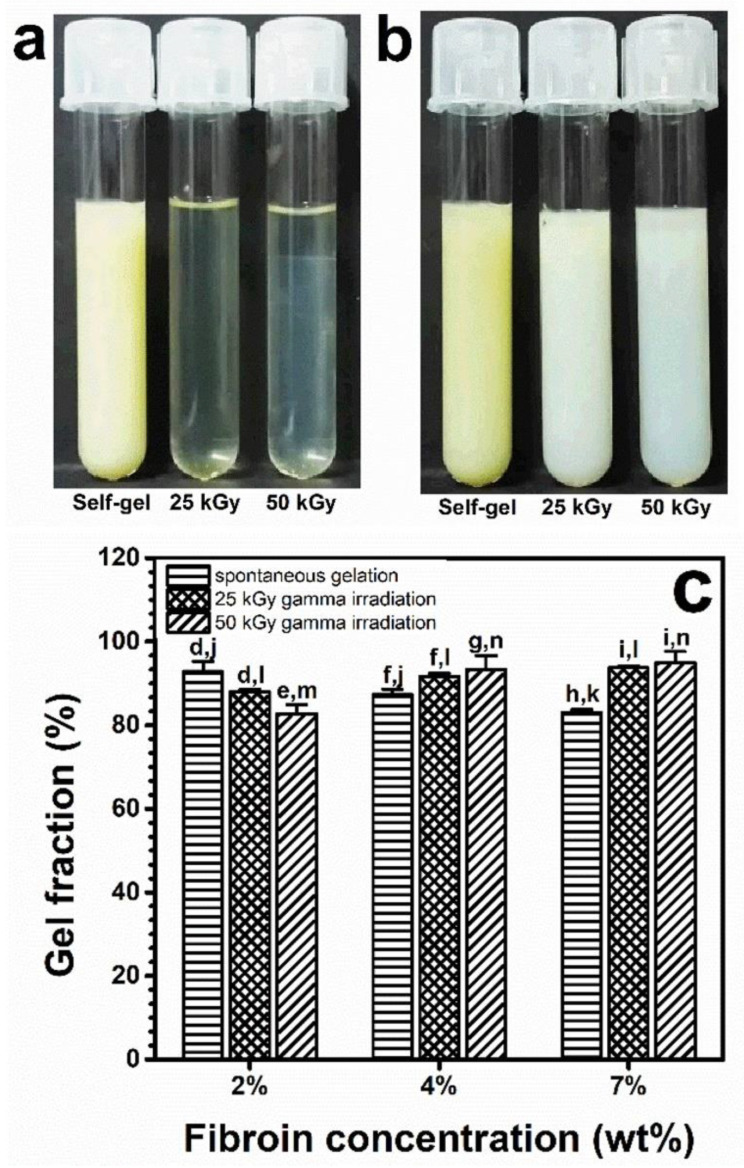
Appearance of SF hydrogels from a 2-weight-by-percentage solution immediately after spontaneous gelation (Self-gel) and gamma irradiation at 25 kGy or 50 kGy (**a**), as well as after being kept for 4 days at 25 °C (**b**). Insoluble gel fractions at 37 °C of SF hydrogels gelled spontaneously and via 25 or 50 kGy gamma irradiation (**c**) from 2-, 4-, and 7-weight-by-percentage fibroin solutions. Each bar shows a mean with its standard deviation; *n* = 3. d–i and j–n indicate the statistical differences at *p* ≤ 0.05, compared both within groups and between groups under the same conditions.

When exposed to irradiation, gelation was obtained when the SF solution was not less than 2 wt%. The gels formed via gamma irradiation were optically clearer than the turbid yellow gels formed via spontaneous gelation at 25 °C (Figure 1a). It was noted that during gamma irradiation, the temperature could rise to 40 °C. When hydrogels were left for 4 days at 25 °C, the optically clear gels became white, while the spontaneously gelled SF remained unchanged (Figure 1b). The gel fractions of the hydrogels were further investigated and revealed that the insoluble gel fraction was inversely proportional to the SF concentration in the spontaneously formed gels. In the gamma-induced gels, the insoluble gel fraction appeared to be directly proportional to the SF concentration (Figure 1c).

**Figure 2:**
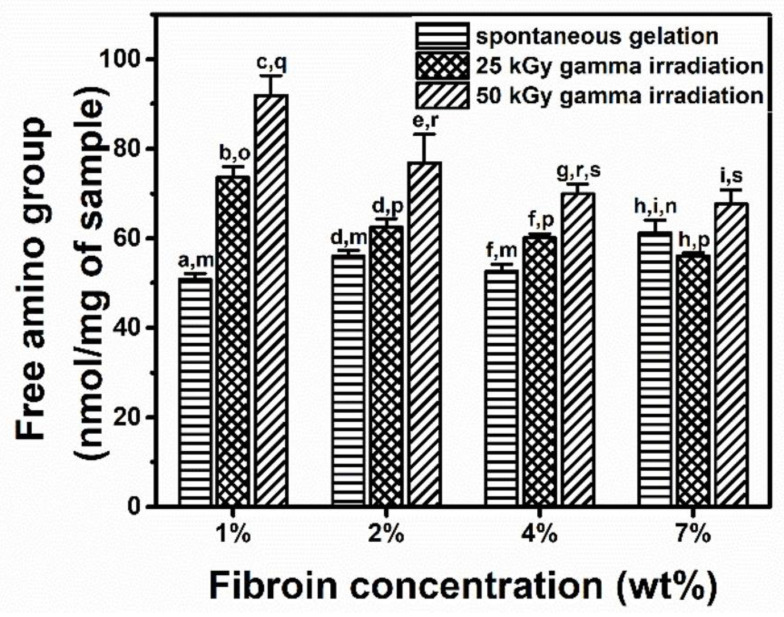
The number of free amino groups present in hydrogels after spontaneous gelation or 25 or 50 kGy of gamma irradiation. a–i and m–s indicate the statistical differences at *p* ≤ 0.05 compared to those within and between groups under the same conditions.

The quantities of free amino groups, which were indicative of polypeptide-chain scission, in the hydrogels were analyzed using the TNBS assay (Figure 2). The number of free amino groups in the gamma-induced hydrogels were significantly greater than those in the spontaneously formed hydrogels. The stronger gamma dose resulted in a greater amount of free amino groups. This finding suggests that there were more polypeptide-chain scissions when a greater gamma dose had been used. Interestingly, the number of free amino groups present in a more concentrated silk fibroin solution was less affected by the gamma dose. This finding might be explained by the dissipation of the irradiation energy. The more concentrated solution underwent less chain scission when exposed to the same amount of irradiation energy as the diluted solution received. It was reported that high-energy radiation can be used to induce polymer gelation [24]. The mechanism via which the radiation promotes gelation was claimed to involve radiation-induced crosslinking by free radicals [24,25].

### 3.2. Thermal Stability

**Figure 3:**
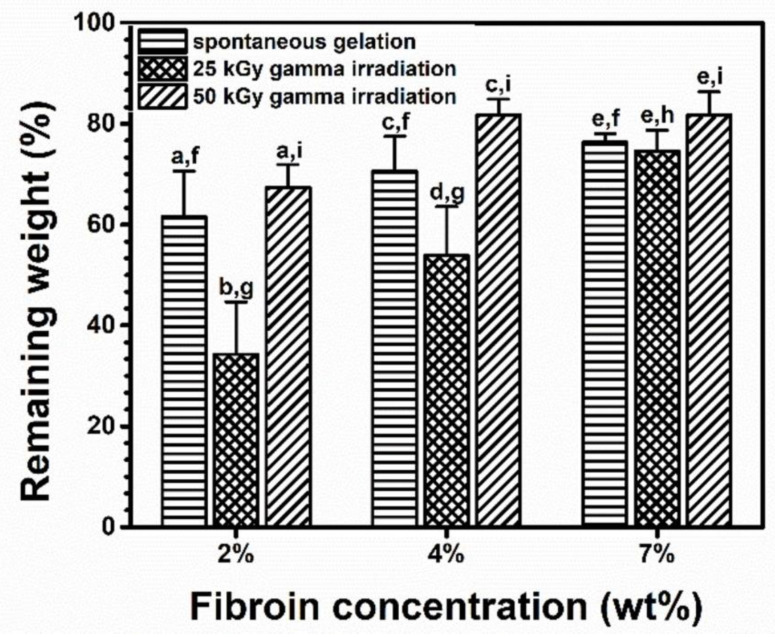
Thermal stability of SF hydrogels formed spontaneously or by 25 or 50 kGy of gamma irradiation from 2-, 4-, and 7-weight-by-percentage fibroin solutions. Each bar shows a mean with its standard deviation; *n* = 3. a–e and f–i indicate the statistical differences at *p* ≤ 0.05 compared to those within and between groups.

The weights of the hydrogels after being heated for three hours at 80 °C were measured. The remaining-weight percentages of the gels formed via spontaneous gelation were not dependent on the fibroin concentration. When the gelation had been induced through gamma irradiation, striking results were found. The remaining-weight percentages of the 25 kGy radiation-induced hydrogels were less than those formed via spontaneous gelation (Figure 3). However, the differences seemed to diminish when the fibroin concentration was increased.

At 80 °C, some of the hydrogen bonds that stabilize the gel could be destroyed. This destabilization resulted in the gels experiencing weight loss. The reason that the remaining-weight percentages of the 25-kilogray irradiation-induced hydrogels were low could be the occurrence of chain scission during the gamma irradiation. The chain scission might randomly cleave the SF chains, rendering them too short to form heat-tolerant structures such as crystalline β-sheets, which is supported by the FTIR result shown in the later section.

Surprisingly, the remaining weight percentages became greater when the radiation dose was raised from 25 to 50 kGy. We think that radiation crosslinking at 50 kGy created a number of covalent crosslinks between the cleaved SF chains, and the covalent crosslinks were more thermally stable than the hydrogen bonds in the crystalline β-sheets. Therefore, we hypothesized that the free radical-mediated crosslinking and the crystalline β-sheet formation acted together to stabilize the hydrogel.

### 3.3. Thermal Responses of the Hydrogel

**Figure 4:**
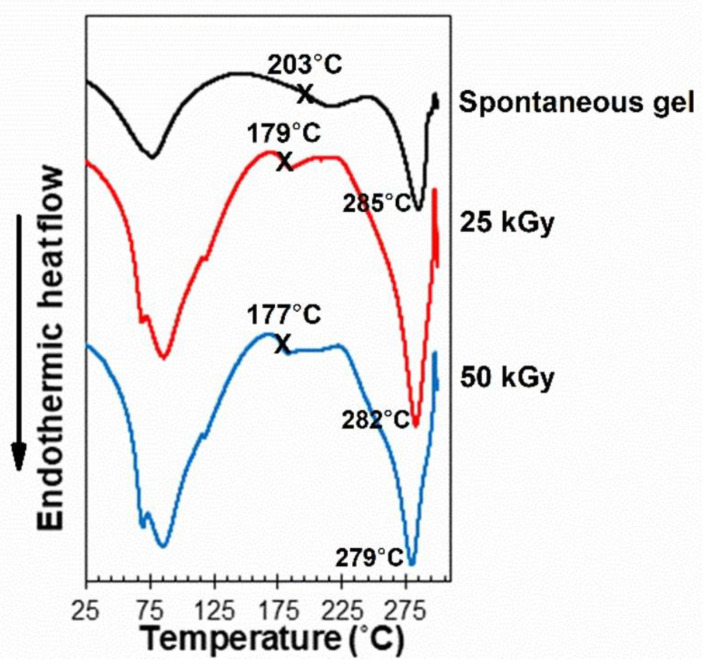
DSC profiles of SF hydrogels formed spontaneously or by 25 kGy or 50 kGy of gamma irradiation from a 7-percentage-by-weight SF solution. The x marks show glass transition temperatures.

The spontaneously formed SF hydrogel made of a 7-weight-by-percentage SF solution showed a broad endothermic peak at 77 °C in the DSC profile, which corresponds to the loss of water from the hydrogel, and a narrower decomposition peak at 285 °C (Figure 4). When the hydrogels had been formed via gamma irradiation at 25 or 50 kGy, the decomposition temperatures (T_d_) of the hydrogels decreased from 285 to 282 and 279 °C, respectively. In addition, a double endothermic peak at 72 °C and 80 °C was observed, implying that different states of water were present in gamma-irradiated hydrogels. Furthermore, the glass transition temperatures (T_g_) of the hydrogels formed spontaneously and via irradiation were different. The T_g_ of the spontaneously formed hydrogels was 203 °C, whereas the T_g_ values of the hydrogels formed by 25 and 50 kGy of irradiation were 179 and 177 °C, respectively. This finding means that the hydrogels were less thermally stable at high temperatures when the radiation dose was increased. This result supports the prediction that excessive chain scission occurred at a higher radiation dose, reducing the ability to form crystalline β-sheets, thus reducing T_g_ and T_d_.

### 3.4. Chemical Structures of the Hydrogel

**Figure 5:**
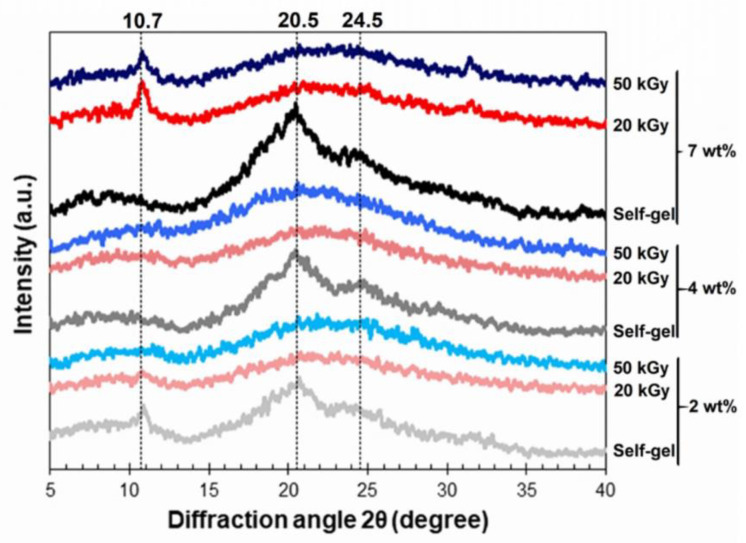
XRD profiles of the SF hydrogels formed spontaneously (self-gel) and by 25 or 50 kGy gamma irradiation from 2-, 4, and 7-weight-by-percentage SF solutions. The dashed lines mark the diffraction angles of 10.7, 20.5 and 24.5 degrees, respectively.

XRD was performed to compare the crystallinity of the hydrogels formed spontaneously or via irradiation. Figure 5 shows that the XRD curves of the spontaneously formed hydrogels exhibited peaks at 10.7, 20.5, and 24.5° corresponding to the crystalline structure of the β-sheets. These peaks are consistent with the previously reported XRD profiles [26]. When the hydrogels had been formed via irradiation, the peaks at those diffraction angles flattened, covering diffraction angles in the range 19–24°. This result implies that the radiation-induced SF hydrogels formed more amorphous structures instead of crystalline β-sheets.

**Figure 6:**
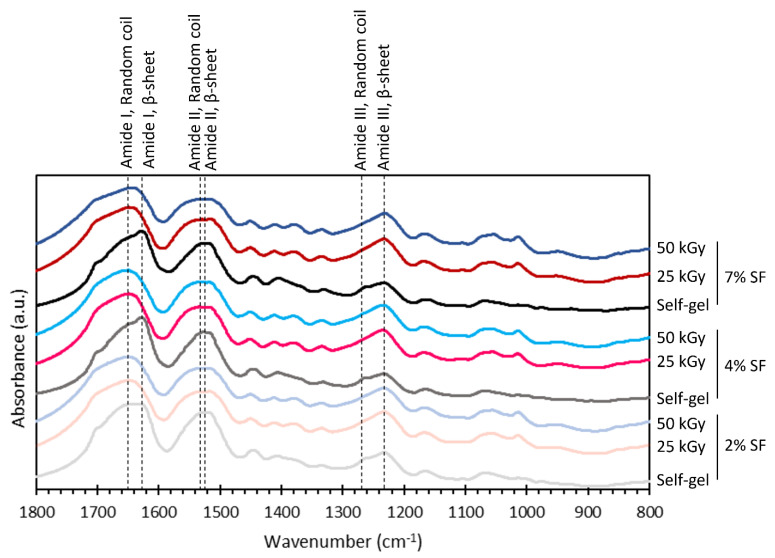
FTIR absorbance spectra of SF hydrogels formed spontaneously (self-gel) and by 25 or 50 kGy of gamma irradiation from 2-, 4-, and 7-weight-by-percentage SF solutions. The dashed lines mark the wavenumbers of 1650, 1627, 1534, 1526, 1270, and 1232 cm^−1^.

As mentioned, we speculated that the changes in the thermal properties and the appearance of the hydrogels formed via irradiation were due to the changes in chemical structures that led to the decrease in the crystalline β-sheets. The XRD results are in good agreement with FTIR results (Figure 6). The spontaneously formed hydrogels had FTIR absorbance peaks at the wavenumbers of 1627 and 1270 cm^−1^, which were assigned to amide I and amide III band ranges of β-sheet structures, respectively [27]. These peaks became subtle in the hydrogels formed via irradiation, while the absorbance peaks at the wavenumbers of 1650, 1534, and 1232 cm^−1^, representing amide I, amide II, and amide III, respectively, of random coils, appeared more clearly [27].

**Figure 7:**
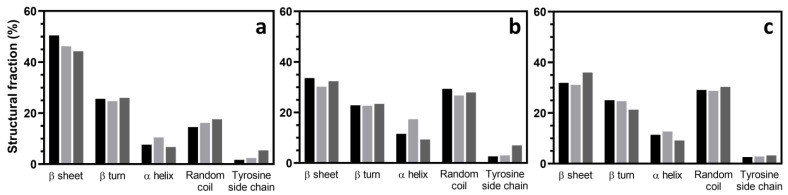
The secondary structures in the SF hydrogels obtained via the deconvolution of the amide I absorbance peak derived from FTIR spectra. The hydrogels were formed via (**a**) spontaneous gelation, (**b**) 25-kilogray of irradiation, and (**c**) 50-kilogray irradiation of the SF solutions with concentrations of 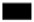 2 wt%, 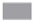 4 wt%, and 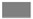 7 wt%.

To extend this analysis, FTIR curve deconvolution of the amide I region between 1575 and 1725 cm^−1^, as shown in Figure S1 in the Supplementary Materials, revealed that the β-sheet fractions decreased when the hydrogel was formed via gamma irradiation (Figure 7). On the other hand, the random coils and α-helices appeared to increase, while β-turns experienced negligible change. When the SF concentration was increased, the same trend was seen. The structural fractions from the deconvoluted FTIR results are consistent with those of the XRD results. This result indicated that gelation initiated via gamma irradiation did not induce the formation of β-sheets as much as it did in spontaneous gelation.

**Figure 8:**
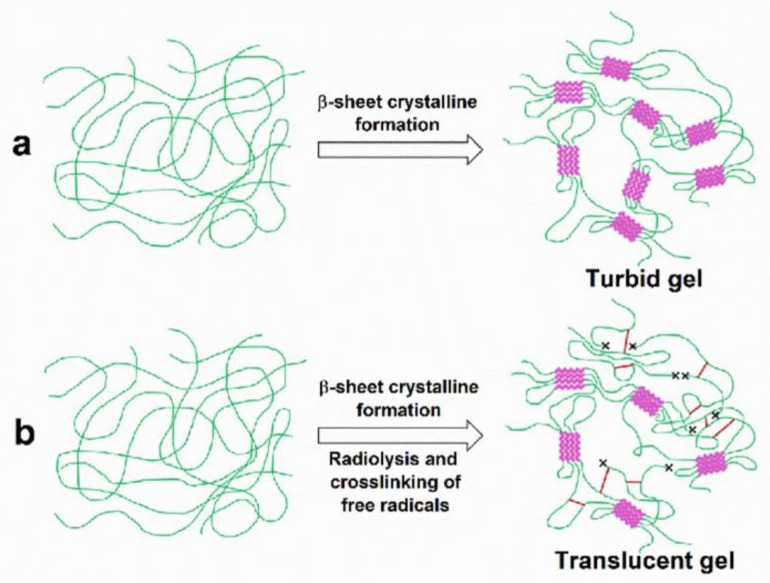
Schematic model of the proposed mechanisms of SF gelation. (**a**) Spontaneous gelation of SF solution is caused by the rearrangement of SF chains (green lines) to form crystalline β-sheet structures (magenta zigzag blocks). The other parts remain in the amorphous state. (**b**) The gamma irradiation-induced gelation of SF solution provides another mechanism to compete with crystalline β-sheet formation. The mechanism involves chain scission and radiation crosslinking by free radicals. The x-marks at the end of SF chains represent breakages of the chains via chain scission. Red lines are crosslinks between the SF chains mediated via the crosslinking reactions of the free radicals.

We believe that the irradiation-induced crosslinking outcompeted the β-sheet formation, as the crosslinking limited the chain flexibility that allowed the β-sheet formation. Therefore, the gelation may not only be caused by the β-sheets, but also by random coils, α-helices and interchain crosslinking. Similar findings were reported in fibroin hydrogels formed via electrogelation [7,24]. In those experiments, the α-helix fraction was increased, whereas the β-sheet fraction was slightly decreased. The proposed mechanism via which the gamma irradiation induces the gelation of SF is illustrated in Figure 8. While the SF solution can spontaneously gel when left at room temperature, gamma irradiation may provide a competing mechanism involving the chain scission of the SF chains. This outcome leads to the breakage of the SF chains and possibly introduces free radicals into the polymers. The free radicals then interact to crosslink SF chains, helping them to quickly form into hydrogels. In addition, gamma radiation heats up the SF solution. The increased temperature generated during irradiation could also affect gelation. Matsumoto et al. reported a quicker gelation of SF solution compared to the that occurring at a lower temperature [5]. Therefore, such combined mechanisms could result in hydrogels formed with distinct characteristics e.g., thermal stability and transition temperatures, depending on the concentration of the fibroin solution and gamma radiation dose.

## 4. Conclusions

This work demonstrates the hydrogelation of a regenerated SF solution using gamma irradiation. The gamma irradiation could induce the gelation of the SF solution when the concentration was not less than 2 wt%. Unlike spontaneous gelation, the gamma irradiation-induced hydrogels had a decreased β-sheet fraction, whereas the fractions of random coils and α-helices were increased. The gamma irradiation resulted in hydrogels that were optically clearer than the spontaneously formed hydrogels. The gamma irradiation-induced hydrogels were thermally stable at body temperature, and the thermal stability could be enhanced when the SF concentration or the radiation dose was increased. We believe that the gamma-mediated chain scission and crosslinking were responsible for generating the hydrogel properties that were different to those of the spontaneously formed hydrogels. This work also shows that gamma irradiation can possibly be a chemical-free choice for the simultaneous fabrication and sterilization of medical hydrogels, reducing the number of preparation steps and, hence, the cost.

## Figures and Tables

**Table 1:** Gelation of silk fibroin solution at various concentrations after gamma irradiation vs. spontaneous gelation at 25 °C.

Fibroin Concentration (wt%)	Spontaneous Gelation at 25 °C	25 kGy	50 kGy
1%	Turbid gel	No gel	No gel
2%	Turbid gel	Translucent gel	Translucent gel
4%	Turbid gel	Translucent gel	Translucent gel
7%	Turbid gel	Translucent gel	Translucent gel

## Data Availability

The data presented in this study are available on request from the corresponding author.

## Supplementary Materials

The following supporting information can be downloaded at: https://www.mdpi.com/article/10.3390/polym15183734/s1, Figure S1: FTIR absorbance spectra of SF hydrogels zoomed to the wavenumber of 1550–1750 cm^−1^. The curves are magnified from Figure 6.
