# Supplementary material for: Hydrogelation of Regenerated Silk Fibroin via Gamma Irradiation
Source: Polymers (Basel). 2023 Sep 12;15(18):3734. doi: 10.3390/polym15183734 (PMC10535586; doi:10.3390/polym15183734)
Supplement: Supplementary file 1 [file polymers-15-03734-s001.zip › polymers-2554414-supplementary.pdf]

Supplementary Material

Hydrogelation of Regenerated Silk Fibroin via Gamma Irradiation

Peerapat Thongnuek [1,2,3], Sorada Kanokpanont [1,4,*], Pimpon Uttayarat [5] and Siriporn Damrongsakkul [1,4]

1 Center of Excellence in Biomaterial Engineering for Medical and Health, Chulalongkorn University,, Bangkok 10330, Thailand; peerapat.t@chula.ac.th (P.T.); siriporn.d@chula.ac.th (S.D.)
2 Biomedical Engineering Program, Faculty of Engineering, Chulalongkorn University, Bangkok 10330, Thailand
3 Biomedical Engineering Research Center, Faculty of Engineering, Chulalongkorn University, Bangkok 10330, Thailand
4 Department of Chemical Engineering, Faculty of Engineering, Chulalongkorn University, Bangkok 10330, Thailand
5 Research and Development Unit, Thailand Institute of Nuclear Technology (Public Organization),
Nakhon Nayok 26120, Thailand; pimponu@tint.or.th
* Correspondence: sorada.k@chula.ac.th

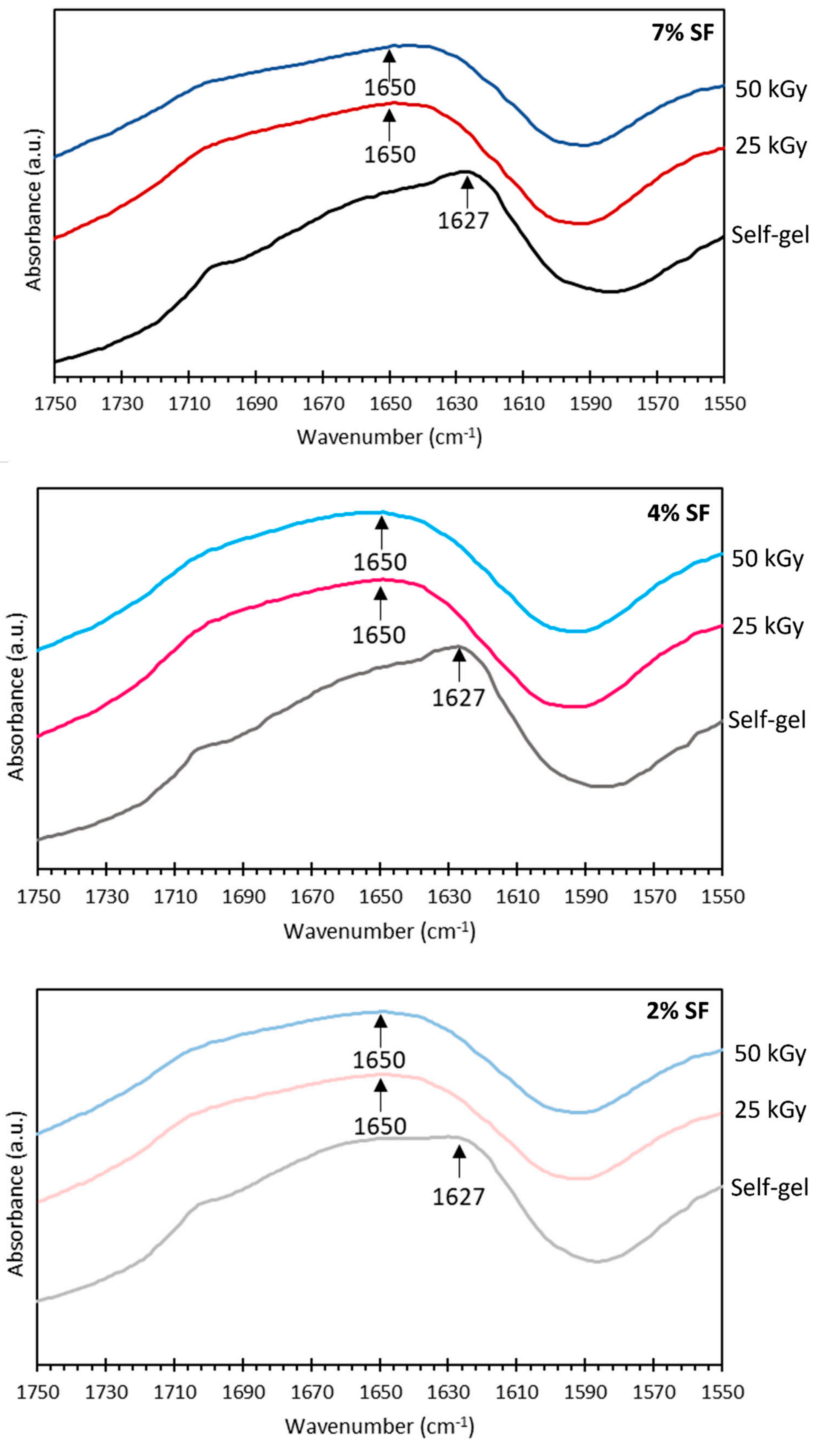

Figure S1 FTIR absorbance spectra of SF hydrogels zoomed to the wavenumber of 1,550 – 1,750 cm^{-1}. The curves are magnified from Figure 6.
